# Identification of putative flowering genes and transcription factors from flower *de novo* transcriptome dataset of tuberose (*Polianthes tuberosa* L.)

**DOI:** 10.1016/j.dib.2018.09.051

**Published:** 2018-09-22

**Authors:** Jayanthi Madhavan, Pawan Jayaswal, Kanchan B.M. Singh, Uma Rao

**Affiliations:** a Division of Nematology, Indian Agricultural Research Institute, New Delhi, India; b National Research Centre for Plant Biotechnology, Pusa, New Delhi 110012, India

**Keywords:** Tuberose, Amaryllidaceae, Transcriptome analysis, KEGG, Flower specific genes, Transcription factors

## Abstract

*Polianthes tuberosa* is commercially popular because of their economic importance in floriculture for cut and loose flowers and in perfume industry because of the unique fragrance. Despite its commercial importance, no ready-to-use transcript sequence information is available in the public database. We have sequenced the RNA obtained from tuberose flowers using the Illumina HiSeq. 2000 platform and have carried out a *de novo* analysis of the transcriptome data. The *de novo* assembly generated 11,100 transcripts. These transcripts represent a total of 7876 unigenes that were considered for downstream analysis. These 7876 unigenes, which was further annotated using blast2go and KEGG pathways, were also assigned. Tuberose transcripts were also assigned to metabolic pathways using the Kyoto Encyclopedia of Genes and Genomes database to determine their biochemical functions. 4591 of the tuberose transcripts matched to genes in KEGG pathways and 66 transcripts were mapped to the Flavonoid biosynthesis pathway. 21 flowering genes have been identified in this tuberose transcriptome. Transcription factor analysis helped in the identification of a large number of transcripts similar to key genes in the flowering regulation network of *Arabidopsis thaliana*. Among the transcription factors identified “NAC” which is associated with plant stress response represented the most abundant category followed by APETALA2 (AP2)/ethylene-responsive element binding proteins (EREBPs) which plays various role in floral organ identity and respond to different biotic and abiotic stress.

**Specifications table**

**Table:** 

Subject area	*Plant Biotechnology and Bioinformatics*
More specific subject area	*Transcriptome*
Type of data	*Table, text file, graph, figure*
How data was acquired	*Illumina Hiseq. 2000 platform at SciGenom Next-Gen sequencing facility*
Data format	*Analyzed*
Experimental factors	*RNA was isolated from flowers of Polianthes tuberosa*
Experimental features	*Transcriptome sequence of tuberose flower and de novo analysis for identification of flowering genes and transcription factors*
Data source location	*New Delhi, India*
Data accessibility	*Data is with this article and the raw sequence data generated has been deposited in the SRA database (*http://www.ncbi.nlm.nih.gov/bioproject/321962*) for public access (BioSample accession ID: SAMN05006898).*

**Value of the data**

• This is the first report of *de novo* transcriptome analysis of *Polianthes tuberosa* flower. Tuberose transcripts were assigned KEGG pathways from the transcriptome data. Flowering genes and transcription factors were identified from the transcriptome data successfully.
• Transcriptome data will provide a strong foundation for research on gene expression, genomics and functional genomics in *Polianthes tuberosa* and other important members of Amaryllidaceae.
• The data generated during this work has not only added so much of information on a plant which had no genomic information on the public domain but also shall help in the studies of other economically important plants like daffodils, snowflakes, onions and garlic belonging to the same family.
• The data will help in the better understanding of expression patterns and their relation to function and regulation, and also the genetic mechanisms, evolutionary relationships between tuberose and other plants.
• This transcriptomic analysis has opened up the prospects for a better understanding of its genomics and we have updated the current gene resource.

## 1 Data

**Fig. 1:**
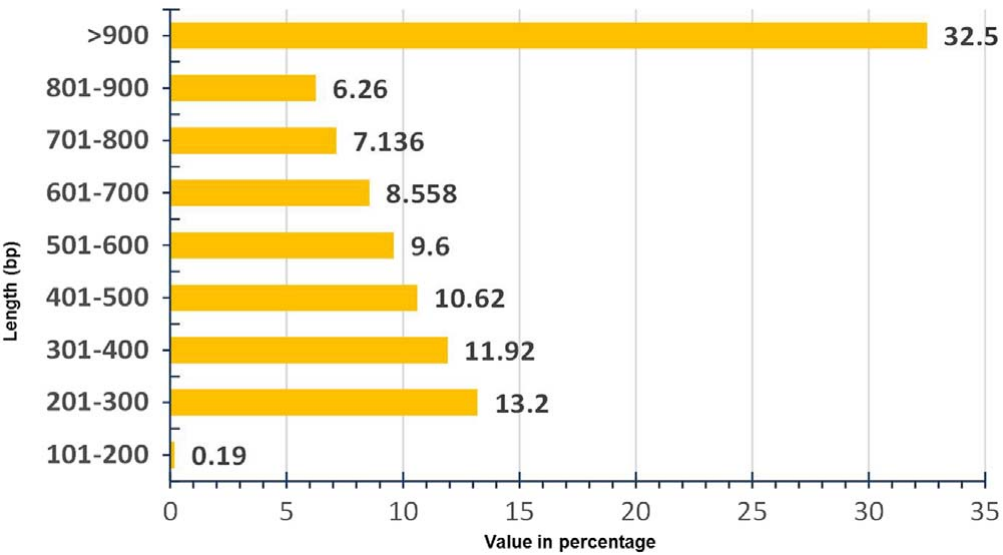
Length distribution of 7876 Unigene sequences.

**Fig. 2:**
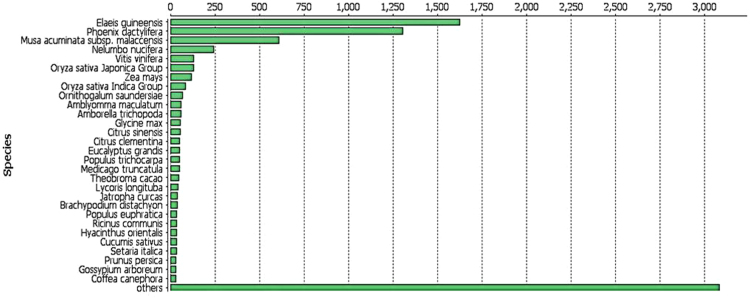
Top BLAST hit species distribution, obtained by BLASTx against the NCBI non-redundant (nr) protein database. The number of top BLAST hits per species is shown on the *x*-axis. Only the 29 most represented species are shown. The complete number of top hits of other organisms is 3080.

In spite of its considerable industrial importance, genomic information on tuberose is very scarce. There are no public Expressed Sequence Tags (EST) or ready-to-use transcripts for *Polianthes tuberosa*. This is for the first time a high-throughput, RNA sequencing (RNA-Seq) of the *P. tuberosa* flower transcriptome was carried out to generate a database that will be useful for further functional analyses. An overview of the sequencing assembly of *P. tuberosa* transcriptome data is presented in Table 1. The length distribution of unigenes is shown in the Fig. 1. The blast result showed that unigenes returned 79.76% (6282) significant hits against the reported datasets. When considering the annotation by species, significant similarity to *Elaeis guineensis* followed by *Phoenix dactylifera* both belonging to the monocotyledons was obtained (Fig. 2).

**Table 1:** Summary of transcriptome sequence assembly of *Polianthes tuberosa* data.

**Content**	**Contig**	**Unigene**
Number	11,100	7876
L50	2692	2000
Minimum length	52	52
N80	511	558
N50	968	1010
N20	1677	1705
Maximum length	9548	9548
Total number of bases	8,238,911	6,236,175

**Fig. 3:**
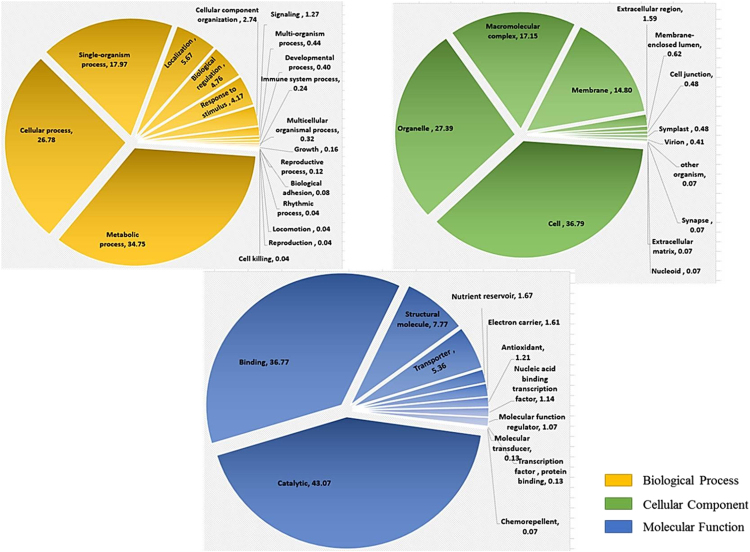
Gene ontology annotations of the 7876 contigs of the *Polianthes tuberosa* transcriptome dataset into three different subcategories like biological process, cellular component and molecular function. Mentioned percentage value indicates the protein-coding *Polianthes tuberosa* transcript assigned to each category.

**Fig. 4:**
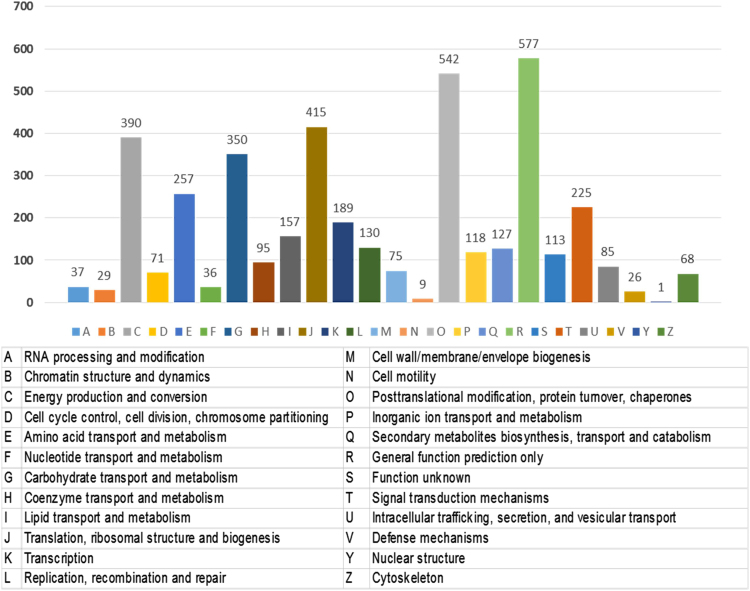
Distribution of clusters of orthologous groups (COGs) of 4122 unigene sequences into 24 different groups.

**Fig. 5:**
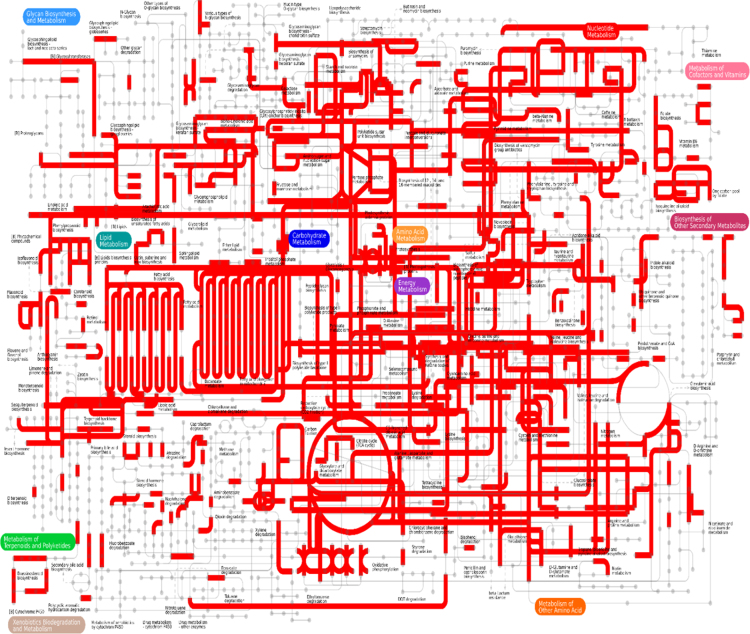
Metabolic pathways active in tuberose as revealed by the transcriptomic analysis using iPATH2 interactive pathway explorer.

**Fig. 6:**
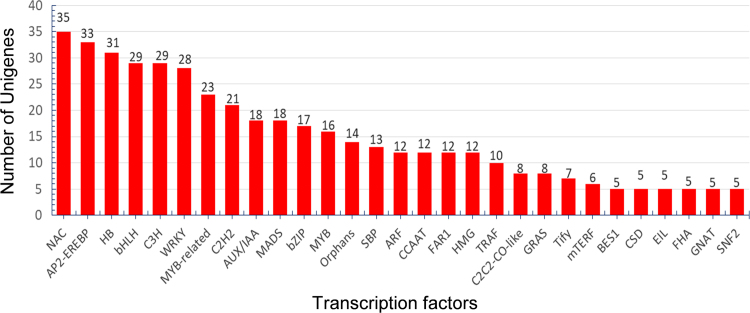
Transcription factor in tuberose distribution of 442 copies (≥5) of TF distributed among 29 different large categories.

Using gene ontology, 1446 ESTs were classified to cellular component category, 2521 ESTs were classified for biological process and 1493 ESTs were classified under molecular function category. A summary with the number and percentage of unigenes annotated in each GO slim term is shown (Fig. 3). According to the data 4122 unique sequences were classified into 24 COG categories (Fig. 4). KEGG Orthology (KO identifiers) for the unigenes were retrieved (Supplementary Table S1a; Fig. 5). As many as 4591 of the tuberose transcripts matched to genes in KEGG pathways (Supplementary Table S1b). We have identified 21 unigenes which showed homology to *Arabidopsis thaliana* flowering genes (Table 2). Analysis of transcription factor in tuberose revealed a total of 511 unigenes, representing 6.48% of the transcriptome classified into 59 putative transcription factors (TF) families (Supplementary Table S2; Fig. 6).

**Table 2:** List of flowering genes homologous to *Arabidopsis thaliana*.

**S.no**	**Tuberose**	**Flowering gene**	**Accession No.**	**Functions**	**References**
1	TUBEROSE_186	NM_114279.4 Ath DNAJ homologue 3 (J3), mRNA	AT3G44110	Flowering promoter; mediates the transcriptional regulation of two floral pathway integrators, FLOWRING LOCUS T and SUPPRESSOR OF OVEREXPRESSION OF CONSTANS 1 and regulates flowering time in *Arabidopsis thaliana*	[8], [9]
2	TUBEROSE_203	NM_118595.5 Ath phosphoglucose isomerase 1 (PGI1), mRNA	AT4G24620	Carbohydrate metabolism, important role in floral initiation, flowering delayed in mutants	[10]
3	TUBEROSE_316	NM_001333000.1 Ath WWE protein-protein interaction domain protein family (RCD1), mRNA	AT1G32230	RCD1–6 mutant showed reduced flowering	[11]
4	TUBEROSE_317	NM_001333000.1 Ath WWE protein-protein interaction domain protein family (RCD1), mRNA	AT1G32230	RCD1–6 mutant showed reduced flowering	[11]
5	TUBEROSE_370	NM_125149.3 Ath CONSTANS-like 5 (COL5), mRNA	AT5G57660	Induce flowering in short day *Arabidopsis thaliana*	[12]
6	TUBEROSE_385	NM_127738.5 Ath cold, circadian rhythm, and RNA binding 2 (GRP7), mRNA	AT2G21660	Promotes floral transition partly by down regulating FLC	[13]
7	TUBEROSE_430	NM_111158.4 Ath GAST1 protein homolog 5 (GASA5), mRNA	AT3G02885	GASA5 is a negative regulator of GA-induced flowering	[14]
8	TUBEROSE_433	NM_001342189.1 Ath homeobox protein ATH1 (ATH1), mRNA	AT4G32980	ATH1 regulates FLC	[15]
9	TUBEROSE_515	NM_130127.2 Ath AGAMOUS-like 6 (AGL6), mRNA	AT2G45650	AGL6 acts as a floral promoter with a dual role, the inhibition of the transcription of the FLC/MAF genes and the promotion of FT expression in Arabidopsis	[16]
10	TUBEROSE_521	NM_001035973.3 AthTransducin family protein / WD-40 repeat family protein (TPL), mRNA	AT1G15750	Represses flowering in *Arabidopsis thaliana*	[17], [18]
11	TUBEROSE_532	NM_001337962.1 Ath ubiquitin-specific protease 13 (UBP13), mRNA	AT3G11910	Control of the circadian clock and photoperiodic flowering	[19]
12	TUBEROSE_589	NM_125149.3 Ath CONSTANS-like 5 (COL5), mRNA	AT5G57660	Induce flowering in short day *Arabidopsis thaliana*	[12]
13	TUBEROSE_589	NM_125149.3 Ath CONSTANS-like 5 (COL5), mRNA	AT5G57660	Induce flowering in short day *Arabidopsis thaliana*	[12]
14	TUBEROSE_597	NM_001344334.1 Ath RNA-binding (RRM/RBD/RNP motifs) family protein mRNA	AT5G40490	HLP1 regulates flowering by alternative polyadenylation	[20]
15	TUBEROSE_645	NM_001332707.1 Athcryptochrome-interacting basic-helix-loop-helix 5 (CIB5), mRNA	AT1G26260	Regulates flowering time redundantly with CIB1.	[21]
16	TUBEROSE_685	NM_102124.3 Ath gigantea protein (GI), mRNA	AT1G22770	promotes flowering under long days in a circadian clock-controlled flowering pathway	[22]
17	TUBEROSE_698	NM_128569.4 Ath UDP-Glycosyltransferase superfamily protein (UGT87A2), mRNA	AT2G30140	Regulates flowering time via the flowering repressor FLC	[23]
18	TUBEROSE_740	NM_114187.5 Ath sucrose synthase 4 (SUS4), mRNA	AT3G43190	Promotes flowering	[24]
19	TUBEROSE_770	NM_101307.5 Ath ubiquitin carrier protein 1 (UBC1), mRNA	AT1G14400	Monoubiquitination of H2B via UBC1 regulates flowering time	[25], [26]
20	TUBEROSE_783	NM_125119.4 Ath Galactose oxidase/kelch repeat superfamily protein (ZTL), mRNA	AT5G57360	Control of flowering time	[27]

## 2 Experimental design, materials, and methods

### 2.1 Plant material

Fully opened tuberose flowers of cultivar Shringar were collected and were immediately frozen in liquid nitrogen and stored at −80 °C.

### 2.2 RNA extraction, cDNA library construction and sequencing

Total RNA was extracted from frozen flower tissues using 596 Nucleospin RNA isolation kit (Macherey-Nagel GmbH & Co. KG, Duren, Germany). Agilent 2100 Bioanalyzer (Agilent Technologies) was used to assess the quality and quantity of RNA. RNA with an RNA integrity number (RIN) of 8.0 was only considered mRNA purification. OligodT beads (Illumina® TruSeq® RNA Sample Preparation Kit v2) were used to purify mRNA from one microgram of total RNA. Elevated temperature (90 °C) in presence of divalent cations was used to achieve the fragmentation of the purified mRNA. cDNA synthesis was done using random hexamers with Superscript II Reverse Transcriptase (Invitrogen Life Technologies). Agencourt Ampure XP SPRI beads (Beckman-Coulter) were used to clean the cDNA. Illumina adapters were ligated to the cDNA molecules after end repair and the addition of an ‘A’ base followed by SPRI clean-up. The resultant cDNA library was amplified using PCR for the enrichment of adapter-ligated fragments, quantified using a Nanodrop spectrophotometer (Thermo Scientific) and validated for quality with a Bioanalyzer (Agilent Technologies). The libraries were then sequenced on Illumina Hiseq. 2000 platform at SciGenom Next-Gen sequencing facility, Cochin, India.

### 2.3 Sequence data assembly and analysis

NGSQC Toolkit version v2.3.3 [1] was used to remove low quality reads (Phred score < 30) and to generate sequencing statistics. High quality paired end filtered reads (15.9 gb) obtained were used for *de-novo* assembly using Velvet (v.1.2.08) and Oases (v.0.2.08) pipeline [2]. Velveth assembly was done with various k-mer range (71- 83) and optimal assembly was attained at k-mer 83. Oases tool was used to identify non-overlapping isoforms/splice variants at minimum transcript length 100. Since our initial target was to identify unique genes. Thus, transcripts were subjected for clustering using CD-HIT-EST [3] 90% similarity. ORF Predictor web server (http://bioinformatics.ysu.edu/tools/OrfPredictor.html) [4] was used to predict proteins from the all non-redundant transcripts (≥100 bp) using the default cut-off value of 1e−5, and 7876 proteins were predicted which were considered for the annotation. The raw sequence data generated has been deposited in the SRA database (http://www.ncbi.nlm.nih.gov/bioproject/321962) for public access (BioSample accession ID: SAMN05006898).

### 2.4 Functional annotation and biological classification of transcripts

Functional annotation of predicted tuberose transcripts was performed using blast2go pipeline on default settings [5]. BLASTP [6] were performed with an *E*-value of 1e−5 to align against NCBI non-redundant (nr) protein database for homology search. Blast results (xml format) were imported to Blast2GO V.3.0.11. GO annotations were performed with default settings and following GO annotation, an Interproscan [7] was performed and results were merged to the GO annotations.

### 2.5 Identification of flowering genes

Homologous flowering gene in tuberose plant were identified using BLASTN programme 306 gene of *A. thaliana* (http://www.phytosystems.ulg.ac.be/florid/) database.

### 2.6 Identification of transcription factors

For the identification of transcription factor in tuberose plant data we used PlnTFDB (3.0) database (http://plntfdb.bio.uni-potsdam.de/v3.0/). Standalone BLASTN programme used for the identification of homologous TF in tuberose plant and output has parsed from BLAST Parser v1.2.6.14 programme (http://geneproject.altervista.org/) and filtered with 60% identity and 100 bit score.
