# Supplementary material for: Identification of putative flowering genes and transcription factors from flower de novo transcriptome dataset of tuberose (Polianthes tuberosa L.)
Source: Data Brief. 2018 Sep 22;20:2027–35. doi: 10.1016/j.dib.2018.09.051 (PMC6174916; doi:10.1016/j.dib.2018.09.051)
Supplement: Supplementary file 1 — Transparency document [file mmc1.docx]

**Title**: Transcriptome analysis of flowers and identification of genes related to flowering and transcription factors in tuberose *(Polianthes tuberosa*)

**The authors declare that there is no conflict of interest.**
